# Transcriptome Analyses of lncRNAs in A2E-Stressed Retinal Epithelial Cells Unveil Advanced Links between Metabolic Impairments Related to Oxidative Stress and Retinitis Pigmentosa

**DOI:** 10.3390/antiox9040318

**Published:** 2020-04-15

**Authors:** Luigi Donato, Concetta Scimone, Simona Alibrandi, Carmela Rinaldi, Antonina Sidoti, Rosalia D’Angelo

**Affiliations:** 1 Department of Biomedical and Dental Sciences and Morphofunctional Imaging, Division of Medical Biotechnologies and Preventive Medicine, University of Messina, 98125 Messina, Italy; 2 Department of Biomolecular Strategies, Genetics and Avant-Garde Therapies, I.E.ME.S.T., 90139 Palermo, Italy; 3 Department of Chemical, Biological, Pharmaceutical and Environmental Sciences, University of Messina, 98125 Messina, Italy

**Keywords:** lncRNAs, oxidative stress, RNA-Seq, retinitis pigmentosa, RPE

## Abstract

Long non-coding RNAs (lncRNAs) are untranslated transcripts which regulate many biological processes. Changes in lncRNA expression pattern are well-known related to various human disorders, such as ocular diseases. Among them, retinitis pigmentosa, one of the most heterogeneous inherited disorder, is strictly related to oxidative stress. However, little is known about regulative aspects able to link oxidative stress to etiopathogenesis of retinitis. Thus, we realized a total RNA-Seq experiment, analyzing human retinal pigment epithelium cells treated by the oxidant agent N-retinylidene-N-retinylethanolamine (A2E), considering three independent experimental groups (untreated control cells, cells treated for 3 h and cells treated for 6 h). Differentially expressed lncRNAs were filtered out, explored with specific tools and databases, and finally subjected to pathway analysis. We detected 3,3’-overlapping ncRNAs, 107 antisense, 24 sense-intronic, four sense-overlapping and 227 lincRNAs very differentially expressed throughout all considered time points. Analyzed lncRNAs could be involved in several biochemical pathways related to compromised response to oxidative stress, carbohydrate and lipid metabolism impairment, melanin biosynthetic process alteration, deficiency in cellular response to amino acid starvation, unbalanced regulation of cofactor metabolic process, all leading to retinal cell death. The explored lncRNAs could play a relevant role in retinitis pigmentosa etiopathogenesis, and seem to be the ideal candidate for novel molecular markers and therapeutic strategies.

## 1. Introduction

In recent years, biomedical research and clinical approaches evolved towards the production, translation and implementation of new technologies and practices. Among them, deep sequencing technologies unveiled that the cellular transcriptional world is far more composite than was initially hypothesized, as the most of genomic sequences could be widely transcribed not only into a very heterogeneous spectrum of protein-coding RNAs, but also in probably wider group of molecular elements made of non-coding RNAs (ncRNAs) [1]. While coding RNAs are abundantly been explored, only a limited range of data is available for ncRNAs. In this scenario, one of the most investigated genetic element is represented by long non-coding RNAs (lncRNAs), a classified group of transcripts longer than 200 nt and up to several kilobases with no apparent protein-coding role [2]. Every form of life is rich of lncRNAs, and specific features of non-coding RNAs, like length or heterogeneity, are strongly linked to organismal complexity [3]. When compared to coding genes, the number of human lncRNAs is much higher, as evidenced by lncRNAs transcription from approximately every locus of the human genome and in different directions [4].

Specifically, lncRNAs could arise from regions overlapping the genomic span (i.e., exon or introns) of a protein-coding locus on the opposite strand (antisense RNAs) or overlapping the 3’UTR of a protein-coding locus on the same strand (3′ overlapping ncRNAs). Moreover, a lncRNA could contain a coding gene in its intron on the same strand (sense overlapping) or could be contained within an intron of a coding gene that does not overlap any exons (sense intronic) [5]. Additionally, several lncRNAs could be transcribed from intergenic regions without overlapping any other known coding gene (lincRNAs) [6] or can derive from the direct ligation of 5′ and 3′ ends of linear RNAs (CircRNAs) or by “backsplicing,”, with a downstream 5′ splice site joined to an upstream 3′ splice site (CiRNAs) [7]. Finally, the most recent lcnRNA subtype, called promoter-associated noncoding RNAs (pancRNAs) or bidirectional promoter RNAs, produced by the divergent transcription from bidirectional gene promoters [8]. The list of characterized lncRNAs is constantly growing, but is already sufficient to highlight their role in gene expression regulation both at a transcriptional and posttranscriptional level [9]. One of lncRNA’s most interesting targets is represented by their host genes, finely regulated in a sequence-specific and structure-specific manner, frequently acting as “sponge” in complex with target microRNAs (miRNAs) [10].

LncRNAs could take part in several critical biological processes such as chromosome conformation modeling, modulation of genomic imprinting, allosteric control of enzymatic activity, as well as coordinating cell state, differentiation and development [11]. Dysregulation or mutation of lncRNA genes has been associated to various human pathologies [12]. Among them, numerous eye–related diseases have already been linked to alteration of lncRNAs, such as ocular tumors, corneal diseases, proliferative vitreoretinopathy (PVR), diabetic retinopathy (DR), retinitis pigmentosa (RP) and other retinal degenerations [13], supported by recent experiments on retinal pigment epithelium (RPE) [14]. An upregulation of the cytoplasmic lncRNA ZNF503-AS1 was consistently revealed during RPE differentiation, while it appeared downregulated in the RPE-choroid of AMD patients [15]. The knockdown of lncRNA-GAS5 was found to maintain the survival of RGCs in glaucoma by activating the TGF-β pathway [16]. LncRNA MIAT was considered as a specific biomarker of age-related cataract (ARC), after evaluation of its high expression both in the plasma and aqueous humor of ARC patients, when compared with other eye diseases [17]. LncRNA-MEG3 resulted down-expressed in STZ-induced diabetic mice and RF/6A endothelial cells treated with hydrogen peroxide and in condition of hyperglycemia [18]. Many lncRNAs, such as BANCR, HOTAIR, DANCR and PANDAR have been found to be positively correlated with retinoblastoma [19], and several others showed pro-angiogenic or anti-angiogenic roles in vascularization in corneal diseases [20]. Thus, the emerging links between non-coding RNAs and pathologies have opened up a new field of diagnostic and therapeutic opportunities [21].

Furthermore, one of main processes involved in the etiopathogenesis of RP is oxidative stress, which could be able to induce, by activation of several cellular degenerative pathways, photoreceptor death [22].

Thus, we analyzed lncRNA expression profiles of a group of RPE cells exposed to the oxidant agent N-retinylidene-N-retinylethanolamine (A2E), one of the best fluorophores of RPE lipofuscin that have been characterized, produced during the process of photoreceptor disk shedding [23]. We realized three independent experiments, considering control group of untreated cells versus cells treated for 3 and 6 h. Principal purpose of our study was to discover which lncRNAs changed during treatment and what their targets are, in order to clarify the biochemical pathways altered by oxidative stress and to identify the ideal candidates for novel molecular markers and therapeutic strategies useful for retinitis pigmentosa.

## 2. Materials and Methods

### 2.1. Cell Culture

Human RPE-derived Cells (H-RPE—Human Retinal Pigment Epithelial Cells, Clonetics™, Lonza, Walkersville, MD, USA) were cultivated in T-75 flasks containing RtEGM™ Retinal Pigment Epithelial Cell Growth Medium BulletKit^®^ (Clonetics™, Lonza, Walkersville, USA) with 2% *v/v* fetal bovine serum (FBS), 1% of penicillin/streptomycin and incubated at 37 °C with 5% CO_2_. H-RPE cells were, then, aliquoted into 96-well plates (4 × 10^4^ cells/well) and grown for 24 h until the confluence prior to the treatment. Afterwards, A2E was added to a final concentration of 20 μM for 3 h and 6 h before rinsing with medium. Control cell groups were incubated without A2E. Reached the confluence, the cultures were shifted to phosphate-buffered saline with calcium, magnesium and glucose (PBS-CMG) and, soon after, exposed for 30 min to blue light produced by a tungsten halogen source (470 ± 20 nm; 0.4 mW/mm^2^) to induce A2E phototoxicity before being incubated at 37 °C.

### 2.2. MTT Assay

Mitochondrial-dependent reduction of methylthiazolyldiphenyl-tetrazolium bromide (MTT) (Sigma-Aldrich, St. Louis, MO, USA) to formazan insoluble crystals was performed to evaluate RPE cell viability during treatment. Briefly, 10 μL of 5 mg/mL of MTT in PBS were added to the cell cultures following the A2E treatment. Following incubation at 37 °C for 2 h, a volume of 100 μL of 10% SDS in 0.01 mol/L HCl was added with the purpose to dissolve the crystals, before being incubated for 16 h. Afterwards, a Dynatech microplate reader permitted to determine the absorbance at 570 nm. Finally, we obtained the percentage of viable cells normalized with control conditions in the absence of A2E. The number of independent experiments was 3.

### 2.3. Total RNA Sequencing

Total RNA was extracted by TRIzol^TM^ Reagent (Invitrogen^TM^, ThermoFisher Scientific, Waltham, MA, USA), following manufacturer’s protocol and quantified at Qubit 2.0 fluorimeter by Qubit^®^ RNA assay kit (Invitrogen^TM^, ThermoFisher Scientific, Waltham, MA, USA). The RNA-seq samples consisted of 3 factor groups, represented by Human RPE cells before the treatment with A2E and at 2 following different time points of 3 h and 6 h, respectively. For each group 3 biological replicates were considered, for a total of 9 samples. Libraries were generated using 1 µg of total RNA by the TruSeq Stranded Total RNA Sample Prep Kit with Ribo-Zero H/M/R (Illumina, San Diego, CA, USA), according to manufacturer’s protocols. Sequencing runs were performed on an HiSeq 2500 Sequencer (Illumina), using the HiSeq SBS Kit v4 (Illumina).

### 2.4. Quality Assessment and Read Alignment

Obtained raw sequences were filtered to remove low quality reads (average per base Phred score < 30) and adaptor sequences. The quality of analyzed data was checked using FastQC (v.0.11.9) (Babraham Institute, Cambridge, UK) [24] and QualiMap (v.2.2.1) (Max Planck Institute, Munich, Bavaria, Germany) [25], while trimming was realized by Trimmomatic (v.0.39) (Usadel Lab, Aachen, Germany) [26]. Filtered data were, then, mapped by Qiagen CLC Genomics Workbench v.20.0 (Qiagen, Hilden, Germany) [27] against the Homo sapiens genome hg38 and the Ensembl RNA database v.99 (EMBL-EBI, Hinxton, Cambridgeshire, UK). Transcriptome analysis was performed using the following settings: quality trim limit = 0.01, ambiguity trim maximum value = 2, mismatch cost = 2, insertion and deletion costs = 3, minimum length fraction and minimum similarity fraction = 0.8, maximum number of hits for a read = 10, strand specific = both.

### 2.5. Gene Expression Quantification and Normalization

Mapped reads were quantified by alignment-dependent expectation-maximization (EM) algorithm [28], particularly useful when most reads map equally well to multiple genes or transcripts. Once the algorithm has converged, every non-uniquely mapping read was assigned randomly to a particular transcript according to the abundances of the transcripts within the same mapping. The transcript per million reads (TPM) values were, then, computed from the counts assigned to each transcript, after normalization by the trimmed mean of M-values (TMM) method [29].

### 2.6. Filtering and Annotation of Non-Coding RNAs

Long non-coding RNAs were filtered from the whole RNA-Seq data, basing on sequence length (reads > 200 nt) and on minimum sampling normalized count (set at TPM ≥ 1). The extracted lncRNA pool was, then, enriched by comparing the tag sequences with the annotation resources UCSC non-coding [30], Ensembl non-coding RNA database v.99 [31], GENCODE v.33 [32], LNCipedia v.5.2 [33], RNAinter [34], RNALocate [35], MNDR v.2.0 [36], ncRDeathDB v.2.0 [37], Malacards and Genecards [38], HUGO Gene Nomenclature Committee (HGNC) expert database [39], lncBase [40], lncRNADisease v.2.0 [41] and lncRNA2Target v.2.0 [42]. All databases provided resources both from experimental and predicted experiments regarding lncRNAs and their targets.

### 2.7. Long Non-Coding RNAs Alignment-Free Algorithms of Analysis

Previously described approaches are alignment-based, either applying multiple alignments to compute the conservation score or pairwise homology search for similar proteins. However, in order to avoid the limitations of alignment-based methods, the most reliable alignment-free algorithms have been applied. Among them, Coding-Potential Assessment Tool (CPAT) [43] and lncScore [44] discriminated non-coding transcripts from protein-coding by logistic regression model as machine learning classifier adopting sequence-based features such as open-reading frame (ORF) length, ORF coverage, ORF size, GC content, hexamer and Fickett scores. CNCI catalogued lncRNAs analyzing adjoining nucleotide triplets (ANT) to detect most-like CDS (MLCDS) regions in each transcript [45]. PLEK uses SVM classifier from LIBSVM package to calibrate k-mer frequencies of a sequence and sliding-window approach as features for classification [46]. Finally, FEELnc [47] accurately annotated lncRNAs using a Random Forest model trained with general features such as multi k-mer frequencies (from k = 1 to 12) and relaxed open reading frames.

### 2.8. Specific Circular RNAs Detection Pipelines

To detect CircRNAs in RNA-seq data an analysis of sequence reads spanning the back-splice junctions produced during circRNAs biogenesis is needed. Back-splice reads align to the genome in chiastic order, so circRNA detection from RNA-seq reads needs specific methods for non-collinear read mapping and analysis. To obtain a reliable result on circRNAs detection, several different methods were applied. The first, a “pseudo-reference-based” tool called KNIFE [48], directly constructed all the putative out-of-order exon–exon junction sequences from gene annotation data before alignment. Next, CIRCexplorer [49] and UROBORUS [50] followed the “fragmented-based” approach, which identified backsplicing junctions from the mapping information of a multiple-split read’s alignment to the genome. Finally, CIRI [51] exploited its own algorithm based on paired chiastic clipping (PCC) signals detection, with an important reduction of potential false positives. Instructions provided in each tool manual were followed, filtering circRNAs with ≥2 back-spliced junction reads.

### 2.9. Differential lncRNAs Expression and Statistical Analysis

Differential expression analysis of lncRNAs was realized using Limma R package [52]. General linear models were applied to compare lncRNA expression changes at the different conditions of experimental design, setting the contrast groups as 0 h.untreated versus 3 h.treated, 0 h.untreated versus 6 h.treated, 3 h.treated versus 6 h.treated, [(0 h.untreated + 3 h.treated)/2] – [(0 h.untreated + 6 h.treated)/2]. The latter came from multiple group mean comparison, that allowed to indirectly capture the differences in expression level determined by the whole period of treatment, hereafter called “Due to Time” effect. For differentially expressed lncRNAs, the log_2_ fold change (log_2_FC) of lncRNA abundance was calculated based on contrast groups and significance of expression changes was determined using the t-test [53]. *p*-values of multiple testing were, then, adjusted with Benjamini & Hochberg (BH) to correct false discovery rate (FDR) [54]. The lncRNAs uniquely identified in the RPE cells, showing at least 3 unique gene reads, lower than two-fold (log_2_FC < –1, down-regulated) or greater than two-fold (log_2_FC > 1, up-regulated) expression changes based on expression values ratio, and with BH–adjusted *p*-values lower than 0.05, were selected for functional classification of differentially expressed lncRNAs.

### 2.10. lncRNAs Validation by qRT-PCR

We selected the ten most dysregulated lncRNAs, obtained from RNA-seq data, to be validated by quantitative Real-Time polymerase chain reaction (qRT-PCR). Reverse transcription was performed following the manufacturer’s protocol of GoScript™ Reverse Transcription System (Promega, Madison, WI, USA). The retrotranscribed cDNA was subjected to the qRT-PCR in the ABI 7500 fast sequence detection system (Applied Biosystems, Foster City, CA, USA), using the BRYT-Green based PCR reaction. PCR amplification was performed in a total reaction mixture of 20 μL, consisting of 20 ng cDNA, 10 μL 2 × GoTaq1qPCR Master Mix (Promega, USA) and 0.2 μM of each primer. The reaction was completed with the standard thermal cycle conditions applying the two-step qRT-PCR method: an initial denaturation at 95 °C for 30 s, followed by 40 cycles of 30 s at 95 °C and 30 s at 60 °C. Each reaction was replicated six times, considering all analyzed time points (18 samples), and the average threshold cycle (Ct) was calculated for each replicate. The lncRNAs expression was normalized to the expression level of most stable reference lncRNA, after being identified as combination of Delta Ct [55], GeNorm [56], NormFinder [56] and BestKeeper [57] algorithms results. The relative gene expression was, then, calculated using the 2^–ΔΔCt^ method, and the results were shown as the mean ± SEM (Standard Error of Mean). Statistical significance was determined by analysis of variance between groups (ANOVA), followed by Bonferroni post-hoc test. Finally, a linear regression analysis was performed to check the correlation of the FC of the gene expression ratios between qRT-PCR and RNA-Seq. The statistical analyses were all performed using IBM SPSS 26.0 software (IBM Corp, Armonk, NY, USA) [58]. Adjusted *p*-values < 0.05 were considered as statistically significant. The research was approved by the Scientific Ethics Committee of the Azienda Ospedaliera Universitaria-Policlinico “G. Martino” Messina.

### 2.11. lncRNA Host and Target Genes Pathway Analysis

GO term enrichment analysis for the most altered lncRNA host and target genes was performed using the ClueGO (v. 2.5.6) (INSERM, Paris, France) [59] and CluePedia (v. 1.5.6) (INSERM, Paris, France) [60] plugins in Cytoscape (v. 3.7.2) (National Institute of General Medical Sciences, Bethesda, MD, USA) [61]. Default parameters were used, except for Min#/% Genes = 40 and K-score threshold = 0.7, due to the huge number of nodes and edges deriving from analysis. Finally, only GO terms with *p* < 0.01 were selected.

### 2.12. Pathway Analysis of microRNA Targeting to Most Altered RPE Expressed lncRNAs

To evaluate the crosstalk between lncRNAs and miRNAs, we exploited the computational resource DIANA TOOLS mirPath v.3.0 (University of Thessaly, Thessaly, Greece) [62]. This bioinformatic web-server platform permitted us to obtain accurate statistics for possible pathways involving miRNAs targeting most dysregulated lncRNAs.

## 3. Results

### 3.1. MTT Cell Viability Assay Showed an Exposure Time-Related Increased Death

**Figure 1:**
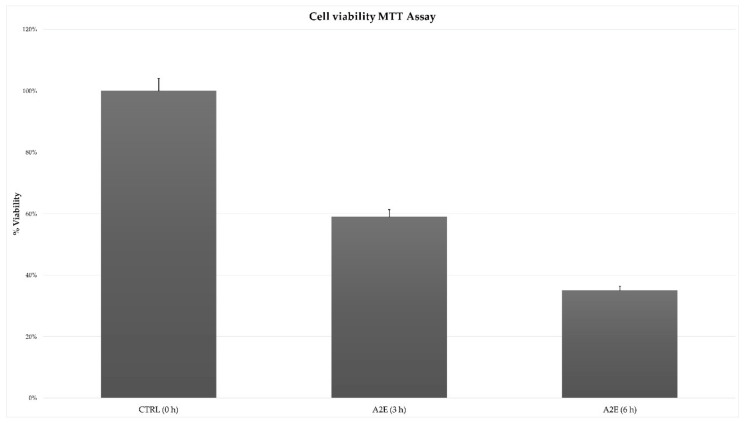
Effect of N-retinylidene-N-retinylethanolamine (A2E) on retinal pigment epithelium (RPE) cells toxicity. Results of MTT assay showed a time-dependent cytotoxicity induced by A2E treatment. Cell viability percentage is expressed as mean ± SD (*n* = 3). Multiple *t*-tests were performed for statistical comparisons (*p*-values < 0.05).

The MTT cell viability assay highlighted a significant and different trend in RPE treated cells versus control. The addition of A2E to cultures led to a decreased percentage of cell viability, with the lowest peak at 6 h after treatment (Figure 1).

### 3.2. Sequencing and Differential Expression Analyses Highlighted a Prevalence of Down-Regulated lncRNAs upon Up-Regulated Ones

**Figure 2:**
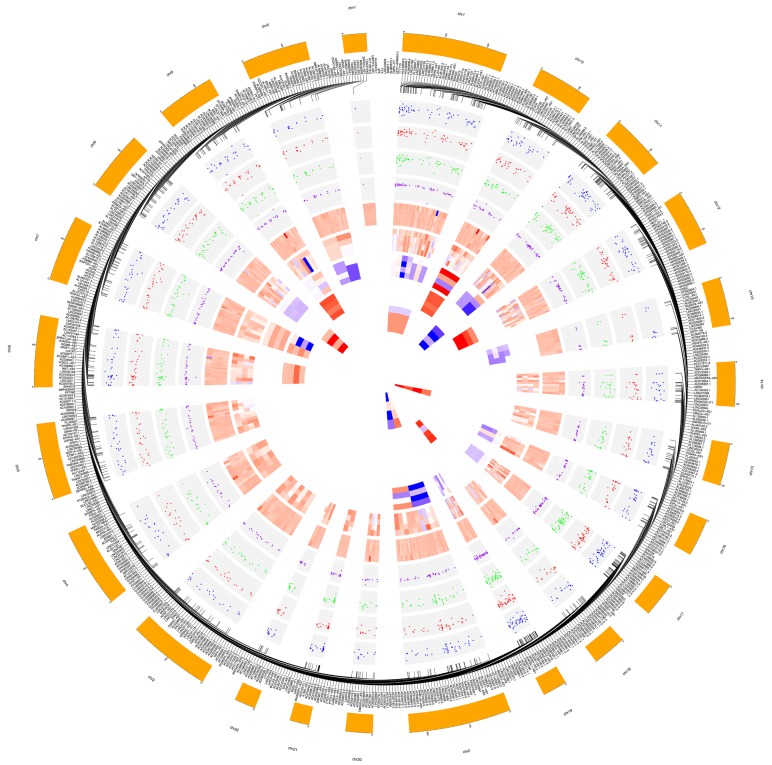
Circular plot made by CIRCOS [63] highlighting fold changes of significant dysregulated long non-coding RNAs (lncRNAs). Chromosome lncRNA host/target genes belong to and most dysregulated lncRNAs are labeled in the two outermost circles. Then, from outside to inside: dispersion plots of log_2_FC of all significant (*p* < 0.05) different expressed lncRNAs in relationship to the whole treatment period (Due_to_Time), to the first considered time point vs basal time (3 h_vs_0 h), to the second time point vs basal time (6 h_vs_0 h) and to the second vs the first treatment time points (6 h_vs_3 h); heat maps for individual subtypes of significant (*p* < 0.05) differentially expressed lincRNAs, antisense, sense_intronic, sense_overlapping, bidirectional_promoter, 3′_overlapping, one type in relation to the mean of all contrast groups (Due_to_Time, 3 h_vs_0 h, 6 h_vs_0 h and 6 h_vs_3 h) for each circle. Larger width means greater fold-change.

RNA sequencing carried out on Illumina HiSeq 2500 yielded a total of about 100 million quality reads (mean mapping quality = 29) and with a percentage of ~70% uniquely mapped. A total of 2313 lncRNAs were identified out of 48,437 reference counterparts, considering the whole human transcriptome annotations and alignment-free detection algorithms. All previous mapping statistics were based on average values calculated for all three replicates in each time point. All 2313 detected lncRNAs were classified in 3′_overlapping ncRNAs (5), antisense (1228), bidirectional_promoter (7), lincRNAs (966), sense_intronic (74), sense_overlapping (31) and circRNAs (2) (Table S1). Variability was significant across samples (*p* < 0.05). Among previously cited lncRNAs, 672 showed expression alterations in evaluated time points, of which 365 highly differentially expressed. In detail, three 3′_overlapping ncRNAs, 107 antisense, 227 lincRNAs, 24 sense_intronic and four sense_overlapping resulted significantly over– (log_2_FC > 1) or under– (log_2_FC < −1) expressed (Figure 2). The global trend foresaw a prevalence of down-regulation (two 3′_overlapping ncRNAs, 74 antisense, 174 lincRNAs, 20 sense_intronic and three sense_overlapping) upon up-regulation (one 3′_overlapping ncRNAs, 33 antisense, 53 lincRNAs, four sense_intronic and one sense_overlapping). Very interesting, the highest differential expressed lncRNAs belonged to only four of all clustered subgroups. Specifically, the top down-regulated lncRNAs were antisense RNAs and sense_overlapping ncRNAs (AL110504.1, log_2_FC = −11.11; AL160408.2, log_2_FC = −9.32; AP003396.1, log_2_FC = −8.59; AC007192.2, log_2_FC = −6.28; AC078909.1, log_2_FC = −6.11; AC105052.4, log_2_FC = −6.10). The top up-regulated, instead, resulted one sense_intronic lncRNAs (AL158166.1, log_2_FC = 5.01), one antisense RNA (SAPCD1-AS1, log_2_FC = 4.70) and two lincRNAs (AL589843.1, log_2_FC = 4.30; AL356056.3, log_2_FC = 4.30). Details about the whole lncRNA differential analysis and annotation enrichment are available in Tables S2 and S3, respectively.

### 3.3. Lnc-RNAs Validation by qRT-PCR

**Figure 3:**
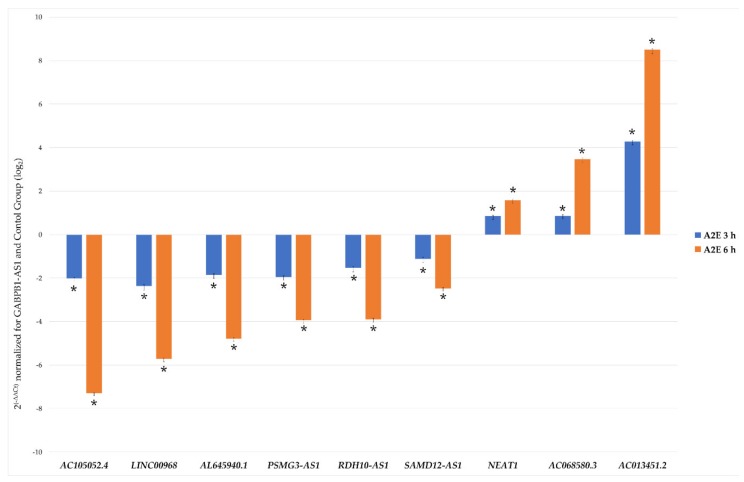
qRT-PCR validation of chosen differentially expressed lncRNAs. Histograms report the mean expression values (*n* of replicate for each group = 6) of nine chosen differentially expressed lncRNAs produced by qRT-PCR experiments, obtained after application of 2^–ΔΔCt^ method, normalized to the best stable lncRNA GABPB1-AS1 (not shown) and control group. Obtained results were statistically significant (ANOVA Bonferroni-corrected * *p*-values < 0.01).

In order to validate the reliability of the RNA-Seq outputs, 10 among the most dysregulated lncRNAs (AC105052.4, LINC00968, AL645940.1, PSMG3-AS1, RDH10-AS1, SAMD12-AS1, GABPB1-AS1, NEAT1, AC068580.3, AC013451.2) were analyzed by qRT-PCR, and the obtained expression profiles resulted very similar to the transcriptome analysis profile (Figure 3 and Table S4). Primer characteristics are listed in Table 1. Results outputted from 2^-ΔΔCt^ method foresaw the normalization based on control group and the best stable lncRNAs GABPB1-AS1, arisen from geometric mean of individual rankings of the four applied algorithms (Figure S1). The analysis of variance (ANOVA) method, conducted to compare the means among multiple groups, highlighted high significance, also resisted to conservative Bonferroni correction (*p*-values < 0.01). Linear regression analysis showed a significantly positive correlation between gene expression ratios of qRT-PCR and RNA-Seq for each evaluated time point (R^2^ = 0.99, *r* = 0.98), confirming our transcriptomic data validity.

### 3.4. Pathway Analysis of Selected lncRNAs Target and Host Genes Shed Light on Metabolic Pathways Impaired by Induced Oxidative Stress

**Figure 4:**
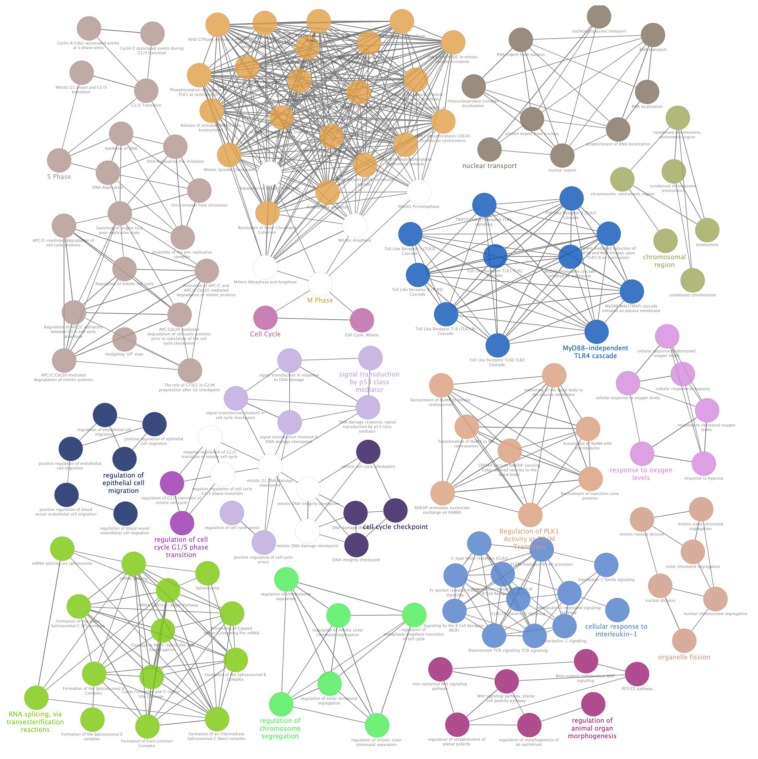
ClueGO enrichment analysis of a first cluster of overrepresented Gene Ontology (GO) terms. The ClueGO plugin of Cytoscape permitted to find a first rich cluster of overrepresented GO processes and a network of connected GO terms was elaborated. Each node represents a GO biological process, and the colors refer to the GO group. Sixteen GO groups are present in the network, one representing GO biological process per group is named in the figure. The edges reflect the relationships between the terms based on the similarity of their associated genes.

**Figure 5:**
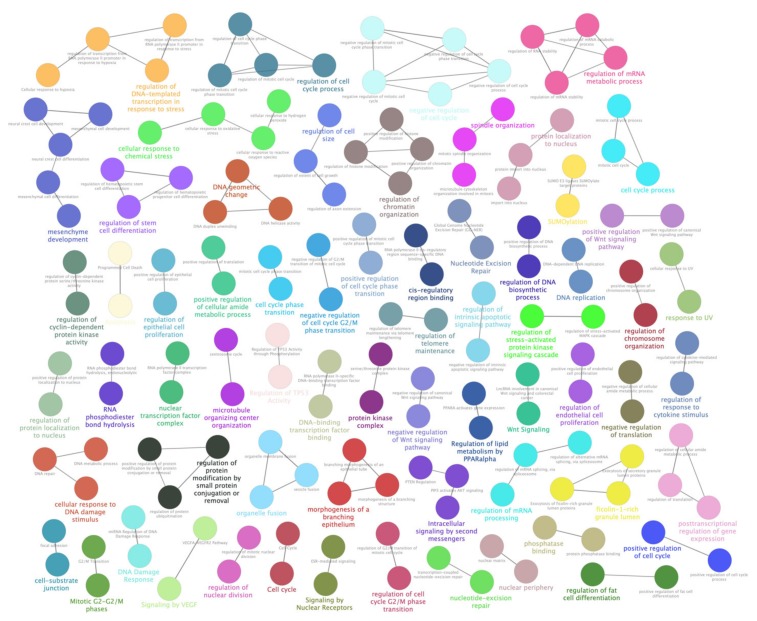
ClueGO enrichment analysis of a second cluster of overrepresented GO terms. The ClueGO plugin of Cytoscape permitted to find a second cluster of overrepresented GO processes and a network of connected GO terms was elaborated. Each node represents a GO biological process, and the colors refer to the GO group. Sixty-four GO groups are present in the network, one representing GO biological process per group is named in the figure. The edges reflect the relationships between the terms based on the similarity of their associated genes.

Most dysregulated lncRNAs were firstly analyzed by RNA Interactome Database (Table S5), and found interactors (including query lncRNAs) were, then, pathway enriched by Cytoscape and its plugins ClueGO and CluePedia. Significant associations emerged between lncRNAs and pathways related to cell cycle alterations, along with cellular response to chemical stress and oxygen levels. However, the most interesting results came from the strong link shown by differentially expressed lncRNAs and metabolism impairments. As evidenced by GO Biological Process and Reactome databases in particular, a huge number of interactors showed high significance (corrected *p*-values near zero) in relationship to their involvement in nucleotides and nucleic acids metabolism (“Metabolism of nucleotides”, 41 interactors; “DNA metabolic process”, 380 interactors; “Positive regulation of DNA metabolic process”, 109 interactors; “Metabolism of RNA”, 276 interactors; “Regulation of mRNA metabolic process”, 190 interactors; “Negative regulation of mRNA metabolic process”, 44 interactors). Additionally, other metabolism-related pathways evidenced strong associations (corrected p-values near zero) with carbohydrate metabolism (“Glucose metabolism”, 40 interactors; “C-type lectin receptor signaling pathway”, 44 interactors; “Insulin signaling pathway”, 55 interactors; “Insulin resistance”, 47 interactors) and lipid metabolism (“Adipogenesis”, 69 interactors, “Regulation of lipid metabolism by PPARalpha”, 53 interactors, “PPARA activates gene expression”, 52 interactors). Furthermore, a novel interesting output of pathway enrichment analysis is related to metabolism of cellular amide (“Regulation of cellular amide metabolic process”, *p*-value = 0.000, 241 interactors; “Positive regulation of cellular amide metabolic process”, *p*-value = 0.000, 92 interactors; “Negative regulation of cellular amide metabolic process”, *p*-value = 0.000, 87 interactors). Finally, two individual regulative pathways, “ABC-family proteins mediated transport” (*p*-value = 0.000, 44 interactors) and “TP53 Regulates Metabolic Genes” (*p*-value = 0.000, 41 interactors), could be also involved in metabolism alterations induced by oxidative stress. Detailed results of most altered pathways and sub-pathways are available in Figure 4 and Figure 5 and Table S6.

### 3.5. LncRNA-miRNA Predicted Interactions Enforced the Hypothesis of RPE Cellular Metabolism Damages Induced by Oxidative Stress

According to LncBase, significant differentially expressed lncRNAs are targeted by 201 miRNAs, and 194 of these were pathway-clustered by mirPath “a posteriori methods” in 12 groups. Among them, eight showed relevant connections with cellular metabolism, involving the analyzed lncRNAs and other genes as interactors: (1) ECM-receptor intersection (*p*-value < 1 × 10^−325^, 28 miRNAs targeting 70 genes); (2) Glycosphingolipid biosynthesis-lacto and neolacto series (*p*-value < 1.09 × 10^−2^, eight miRNAs targeting 13 genes); (3) Mucin type O-Glycan biosynthesis (*p*-value < 1 × 10^−325^, 22 miRNAs targeting 31 genes); (4) Fatty acid biosynthesis (*p*-value < 1 × 10^−325^, nine miRNAs targeting five genes); (5) Fatty acid metabolism (*p*-value < 1 × 10^−325^, 11 miRNAs targeting 16 genes); (6) TGF-Beta signaling pathway (*p*-value < 1.11 × 10^−2^, 12 miRNAs targeting 26 genes); (7) Thyroid hormone synthesis (*p*-value < 8.18 × 10^−4^, 5 miRNAs targeting six genes); (8) Lysine degradation (*p*-value < 7.57 × 10^−4^, eight miRNAs targeting 16 genes). Details on the individual miRNAs belonging to each cluster are available in Table S7.

## 4. Discussion

The relevance of lncRNA genes has been established by their nearness to developmental regulators in the genome, enrichment of tissue-specific and developmental stage-specific expression patterns, and recurrent association with genetic traits [64]. LncRNAs regulate a huge number of molecular and cellular processes, such as epigenetic modifications, RNA splicing, mRNA decay, mRNA translation and molecular scaffold for structural/functional complexes [65]. Together with microRNAs and other small non-coding RNAs, expression alteration of lncRNAs could underlie pathogenesis of a broad range of human diseases [66]. Aside from their well-established roles in cancer [67], lncRNAs are crucial to cardiovascular [68], neurological [69], respiratory [70] and neurodegenerative [71] diseases. Among them, retinitis pigmentosa, an eye–related group of pathologies characterized by very heterogeneous genotypes but quite overlapping phenotypes, shows unusually complex molecular genetic causes, most of which are still unknown [72]. Recently, many experiments showed the important involvement of lncRNAs in retinal disorders, especially regulating angiogenesis, photoreceptor maturation, cell cycle in photoreceptor progenitor cells, apoptosis and cell viability, retinal vessel dysfunction, endothelial cell proliferation, vulnerability of the optic nerve, Muller cell proliferation and neurodegeneration [73]. Although the non-coding scenario of retinal dystrophies is now clearer than before, many obscure sides remain, especially about retinitis pigmentosa specific pathways and the connection between them.

Using recent methods of deep sequencing, we carried out an interesting analysis of the whole transcriptome of RPE cells during a follow-up of two time points (3 h and 6 h) after treatment with A2E. Oxidative stress is considered one of the most significant biochemical pathways involved in RP etiopathogenesis, especially targeting the high metabolic demand of RPE cells. Metabolic dysfunctions (e.g., ceramide synthesis [74]) and impairment of high energy processes as life-long light illumination or physiological phagocytosis could determine pathological modifications in blood-retinal barrier (BRB) and in other extracellular matrix molecules, leading to photoreceptor outer segments (POSs) processing inhibition and RPE cells apoptosis [75,76].

Thus, a deep knowledge of metabolism regulation of RPE cells, along with the identification of links between impairments in metabolic demand and downstream pathways, could shed new light on etiopathogenesis mechanisms of RP.

The realized lncRNA differential expression analysis highlighted a significant impairment in several pathways related to RPE metabolism, starting from nucleic acids. It is well known that lncRNAs are able to be involved in DNA metabolic processes, exerting epigenetic functions by acting as scaffolds for chromatin-modifying complexes [77]. The global up-regulation of DNA metabolic process (involving more than 500 elements between analyzed lncRNAs and their potential interactors) evidenced through observational time-points could determine an increased rDNA silencing, due to chromatin-associated lncRNAs tethered to specific genome sites by forming RNA-DNA hybrids acting in cis, via ATP-remodeling complex NoRC. Such effects could alter cellular growth and synthesis of ribosomes, leading to RPE cell death [78]. Interestingly, two of dysregulated lncRNAs, NEAT1 and MALAT1, are known to be involved in the constitution of paraspeckles nuclear bodies, involved in the trapping of adenosine-to-inosine edited RNA and in retention of serine/arginine (SR) splicing factors [79,80]. MALAT1, whose expression resulted mainly down-regulated after induced oxidative stress, could play its activity on cell survival, especially Muller and ganglion cells (RGCs). Reduced MALAT1 expression may impair neurotrophic factor- and stress-related gene expression by PI3K/Akt, MAPK, Ca2+/CaMK and cAMP/PKA pathways, all converging on the CREB family of leucine-zipper transcription factors [81]. NEAT1, slightly up-regulated during treatment period, could protect retinal Muller cells from apoptosis, also regulating splicing events involving genes related to cell cycle and cell death [82]. The opposite expression trends by MALAT1 and NEAT1 might represent a critical metabolic scenario with a final attempt by impaired cells to survive. Due to the wide spectrum of functions realized by lncRNAs, it is very probable that many of them could arise upon DNA damage, as evidenced by many of detected dysregulated lncRNAs involved in in double-strand break repair, DNA ionizing radiation (IR)-damage and cellular response via ATR, DNA damage and integrity checkpoints, signal transduction in response to DNA damage, also regulated by p53 class mediators. Two of the most interesting lncRNAs probably induced by DNA damage are MNX1-AS1 and MIR31HG, both over-expressed throughout observed time points. These lncRNAs interact with Cyclin D1 (CCND1) mRNA, whose encoding gene is already known to transcribe a specific lncRNA in DNA damage condition, and that exists on chromatin both as RNA:DNA hybrids and ssRNA, acting as transcription repressor [83]. Furthermore, various lncRNAs could exert their role outside the nucleus, hybridizing with the 3′ untranslated regions (3′ UTRs) of mRNAs to regulate their stability in the cytoplasm and/or interacting with miRNAs and RNA decay factors [84]. We found at least 40 lncRNAs, the most of which are antisense lncRNAs, acting as miRNA sponges, and they were mostly down-regulated. However, 26 antisense lncRNAs showed an heterogenous trend: 8 resulted over-expressed and 18 down-expressed. More interestingly, among them, four are the lncRNAs that highlighted the highest fold-change during the observed time points. The two most up-regulated (AC118658.1 and AC145207.2) sponge miRNAs are involved in transcriptional regulation, RNA transport and FoxO/AMPK signaling pathways, probably trying to positive regulate these processes, against the possible transcriptional and mitotic misregulation, along with extracellular matrix alterations, determined by miRNAs interacting with the other two most down-regulated (AL110504.1 and AC078909.1).

As expected, the induced oxidative stress determined changes also in carbohydrate metabolism, particularly in glucose metabolism. About 40 interactors between lncRNAs and their targets/host genes resulted implicated in bioenergetic reactions related to glucose high demand in RPE. In particular, BDNF-AS and TUG1, down-expressed and over-expressed respectively, are already known to affects cell viability by regulating glycolysis [85]. A dysregulation of these two lncRNAs is linked to high-glucose induced (DGI) apoptosis both in RPE and in neuron cells, like other retinal cytotypes [86,87]. Thus, the reduced expression of BDNF-AS and the increased expression of TUG1 could be interpreted as an attempt by RPE cells to protect themselves from apoptosis. Moreover, TUG1 is also an interactor of ARF1 and AKT1, both involved in insulin signaling pathway [88]. Very curiously, other two deregulated lncRNAs, CRNDE (down-expressed) and CYTOR (over-expressed), interact with the already cited ARF1 and AKT1, and with CAP1 and ACACA, also involved in insulin-related pathways [89,90]. Recently, it was experimentally proved that insulin signaling in the RPE may provide a paracrine signal to the retina for maintenance of photoreceptor function and survival, even if partially able to induce oxidative stress [91]. Additionally, high glucose level influences the synthesis of IGF-1, PEDF, advanced glycosylation end (AGE) products and their receptors (RAGE). In particular, it was proved that AGE-RAGE system, activated by glucose-stimulated RPE cells, could determine oxidative stress and inflammatory reactions, ultimately inducing retinal visual cycle impairments [92]. Thus, the combination of both CRNDE and CYTOR dysregulation could enforce the possible protection of RPE cells from DGI by the reduction of glucose intracellular intake and metabolism, as already discussed in relationship with colorectal cancer. Moreover, eight miRNAs, interacting with 10 found dysregulated lncRNAs, could be involved in lacto- and neolacto-glycosphingolipid biosynthesis [93]. Such analyzed lncRNAs showed a global up-regulation, probably silencing sponged miRNAs related to ceramide-ER Stress-AMPK Signaling axis, with the final consequence to induce RPE cell apoptosis.

One of the most investigated metabolic side of the retina regards lipids, mostly phospholipids and vitamin A derivatives. RPE expresses factors required for lipoprotein intake, cholesterol and lipoprotein synthesis and secretion, as well as reverse cholesterol transport (RCT) [94]. Phagocytosis and degradation of the POSs are the principal lipid source for the RPE [95], as well as processing and recycling back to the photoreceptors of vitamin A and of DHA [96,97]. Moreover, excess cholesterol is loaded on HDLs by ABC transporters and, then, removed, or it is also secreted in VLDL-like lipoproteins basolaterally into Bruch’s membrane where it forms drusen [98]. Since HDLs cycle cholesterol between the RPE and photoreceptors, HDLs might accumulate the cholesterol coming out from rod outer segments in the subretinal space, as result of the compromised lipid transport following RPE impairment [99]. In order to perform these tasks, RPE cells present fine-regulated intracellular signaling pathways linking phagocytosis to the transcription of genes involved in lipid metabolism and homeostasis. Such pathways are mainly transcriptionally regulated by the peroxisome proliferator-activated receptor (PPAR) family of nuclear hormone receptors, which act as ligand-dependent transcription factors. This family also includes receptors for retinoids, vitamin D, and thyroid and steroid hormones [100]. The alfa subtype, in particular, stimulates fatty acid catabolism in several tissues known for their high rates of fatty acid oxidation (e.g., liver and eye) [101]. Our experiment highlighted a prevalent down-regulation of identified lncRNAs possibly involved in fatty acids biosynthesis and metabolism. In particular, the down-expression of AC007283.1 (interacting with UPF1 and ELAVL1) and AC012442.2 (interacting with HNRNPC and CSTF2T), along with the over-expression AC089983.1 (the antisense of TXNRD1, interacting with CRNDE) could alter the gene expression and lipid metabolism regulation by PPAR-alfa. Additionally, several down-regulated lncRNAs, such as the antisense AC004943.2 and the sense-overlapping AC007036.3, showed the possibility to interact with various miRNAs involved in fatty acids metabolism and biosynthesis, as well as in thyroid hormone synthesis, depicting a probable silencing scenario which could impair the integrity and functionality of lipidic retinal structures. Very interesting, 44 interactors between identified dysregulated lncRNAs and their targets/host genes resulted involved in ABC-family proteins mediated transport, probably impair the excess cholesterol removal, leading to cell death, especially following the 6h-treatment time point.

Finally, the protein metabolism also showed the involvement of numerous identified lncRNAs. It is well known how misfolding of a huge number of proteins related to retinal survival and vision process, like rhodopsin, can result in disruptions of cellular protein homeostasis [102]. Moreover, it is high probable that the interaction between several chaperones could be altered by oxidant agents, due to activation/deactivation switches that induce certain chaperone-encoding genes or co-chaperones, and repress other ones [103]. We detected two clusters made of dysregulated lncRNAs and their interactors/host genes, involved in positive (92 interactors) and in negative regulation of cellular amide metabolism (87 interactors), respectively. Among them, particularly interesting resulted the up-regulated PTEN-induced putative kinase protein 1 (PINK1) antisense RNA, and the down-regulated FMRP Translational Regulator 1 (FMR1)-IT1 sense intronic and vimentin (VIM) antisense 1 RNA. The first is well known to regulate mitochondrial damage, promote mitophagy and protect cells from death and apoptosis, especially during high glucose mediated regulation of RPE [90]. Thus, the over-expression of PINK1-AS could represent an oxidative stress induced signal reflecting the apoptotic status of treated RPE cells. In the meantime, the down-expression of both FMR1-IT1 and VIM-AS1, related to synaptogenesis, intracellular trafficking and cellular stability [104,105], could be seen as a final attempt of RPE cells to boost the production and sorting of vital proteins towards the most essential cellular districts. Additionally, nine miRNAs interacting with several down-regulated lncRNAs resulted involved in lysine degradation pathway. Several studies have documented N^ε^-Carboxy methyl lysine (N^ε^-CML), one of the most prevalent AGE, as a biomarker of diabetic retinopathy, linked to disruption of retinal photoreceptor external limiting membrane and ellipsoid zone [106]. Therefore, the reduced sponge activity by the previously cited lncRNAs could block lysine degradation, probably trying to arrest the production of AGEs, towards the final purpose to avoid the total cell death.

## 5. Conclusions

Nucleic acids are vital for cell survival, and hence for life. RNA plays innumerable roles in the cell—from gene expression modulation to regulation of protein translation or to control the architecture of whole chromosomes. LncRNAs represent a particular class of molecules characterized by the lack of protein-coding potential but with fundamental features including unique regulatory mechanisms, cis-regulatory activities, alternative forms of biogenesis and functional structured RNA domains. With modern high-throughput RNA-sequencing and advanced epigenomic technologies, the discovery rate of new lncRNA genes is rapidly increasing. We realized a whole transcriptome analysis to evaluate the lncRNA expression changes following the treatment with A2E in RPE cells. About 600 lncRNAs showed significant expression alterations in treated samples, involving target/host genes related to biochemical pathways associated to all major fields of cellular metabolism. Many specific sub-pathways were never linked to mechanisms that underlie retinal degeneration and related pathologies like retinitis pigmentosa. Despite this, a deeper transcriptome sequencing on a wider number of samples could permit to increase the number of detected lncRNAs, further clarifying regulative aspects of these non–coding RNAs in RP etiopathogenesis. Additionally, performing functional assays, (e.g., RNA pull-down, electrophoretic mobility shift assay (EMSA), RNA structure mapping, crosslinking immunoprecipitation (CLIP), phylogenetic lineage tracing, ribosome profiling, etc.) will permit to experimentally confirm the interaction between detected lncRNAs and their interactors. Exploiting innovative techniques, we will surely discover even more intriguing and exclusive features and functions of lncRNAs, improving their link to retinal disease etiopathogenesis.

## Figures and Tables

**Table 1:** List of primers used for qRT-PCR of 10 selected lncRNAs. Table shows features of primers used for qRT-PCR validation of ten selected lncRNAs.

Name	Gene ID (ENSEMBL)	Primer Forward (5′ → 3′)	Primer Reverse (5′ → 3′)	Length (bp)	TM (°C)
*AC105052.4*	ENSG00000279168	GTGTGATAAGATACTGCACTTGG	GGATTTCGCCACGTTGCC	131	61
*LINC00968*	ENSG00000246430	CCACCATCCCATTGAGAACC	TTAGCTGGGAAGGATGAATGC	108	60
*AL645940.1*	ENSG00000272217	TAGGCTTAGGGTGGGTCAGG	TTGTCTGGTGGCAAGATCCC	132	62
*PSMG3-AS1*	ENSG00000230487	GGAAATGTGGGAGGGATGGC	GGGCTCCGACATTGAAGATGG	137	63
*RDH10-AS1*	ENSG00000250295	TGACTACAGCGAGCAACAGC	TCCACTGAGACGGAAACTGC	138	62.5
*SAMD12-AS1*	ENSG00000281641	CAAGGGAGGCAGGACTTTACG	AGTGTCCCTGATGCGAAACG	125	63
*GABPB1-AS1*	ENSG00000244879	TGTCTCATCTCAGTTTCCACAGG	GCAGCACTCTAATCCATCAGC	120	62
*NEAT1*	ENSG00000245532	TCATGAGCGAAGTGAAATTGC	AATAGACGCAGCTCAGAACC	110	60
*AC068580.3*	ENSG00000235027	CGCGCTAGGACAATCAGG	GGAAGCCCAAGACTCACAGG	107	63
*AC013451.2*	ENSG00000258976	CCAACTCAAACCAAATGAAGGG	CCGAGGTGCCTGTAACATCC	126	62

## Supplementary Materials

The following are available online at https://www.mdpi.com/2076-3921/9/4/318/s1, Figure S1: qRT-PCR selected lncRNAs stability results. The funnel chart shows the ranking of analyzed lncRNAs stability, obtained from the geometric mean of rankings values computed by Delta CT, GeNorm, NormFinder and BestKeeper algorithms. The lower stability value corresponds to more stably expressed gene., Table S1: RNA-Seq analysis on the gene level, Table S2: RNA-Seq differential expression analysis of the most dysregulated lncRNAs, Table S3: Annotation enrichment of most dysregulated lncRNAs, Table S4: Fold change expression of selected lncRNAs after A2E treatment, calculated by ΔΔCt method, Table S5: RNAInteractor Database analysis of lncRNAs, Table S6: ClueGo pathway analysis of most dysregulated lncRNAs, Table S7: mirPath analysis of miRNAs sponged by most dysregulated lncRNAs.

## Data Deposition

According to the journal guidelines, RNA-Seq data was deposited in NCBI’s Sequence Read Archive (SRA) and assigned to the project identifier PRJNA622997.
