# Supplementary material for: Transcriptome Analyses of lncRNAs in A2E-Stressed Retinal Epithelial Cells Unveil Advanced Links between Metabolic Impairments Related to Oxidative Stress and Retinitis Pigmentosa
Source: Antioxidants (Basel). 2020 Apr 15;9(4):318. doi: 10.3390/antiox9040318 (PMC7222347; doi:10.3390/antiox9040318)
Supplement: Supplementary file 1 [file antioxidants-09-00318-s001.zip › Figure S1.pdf]

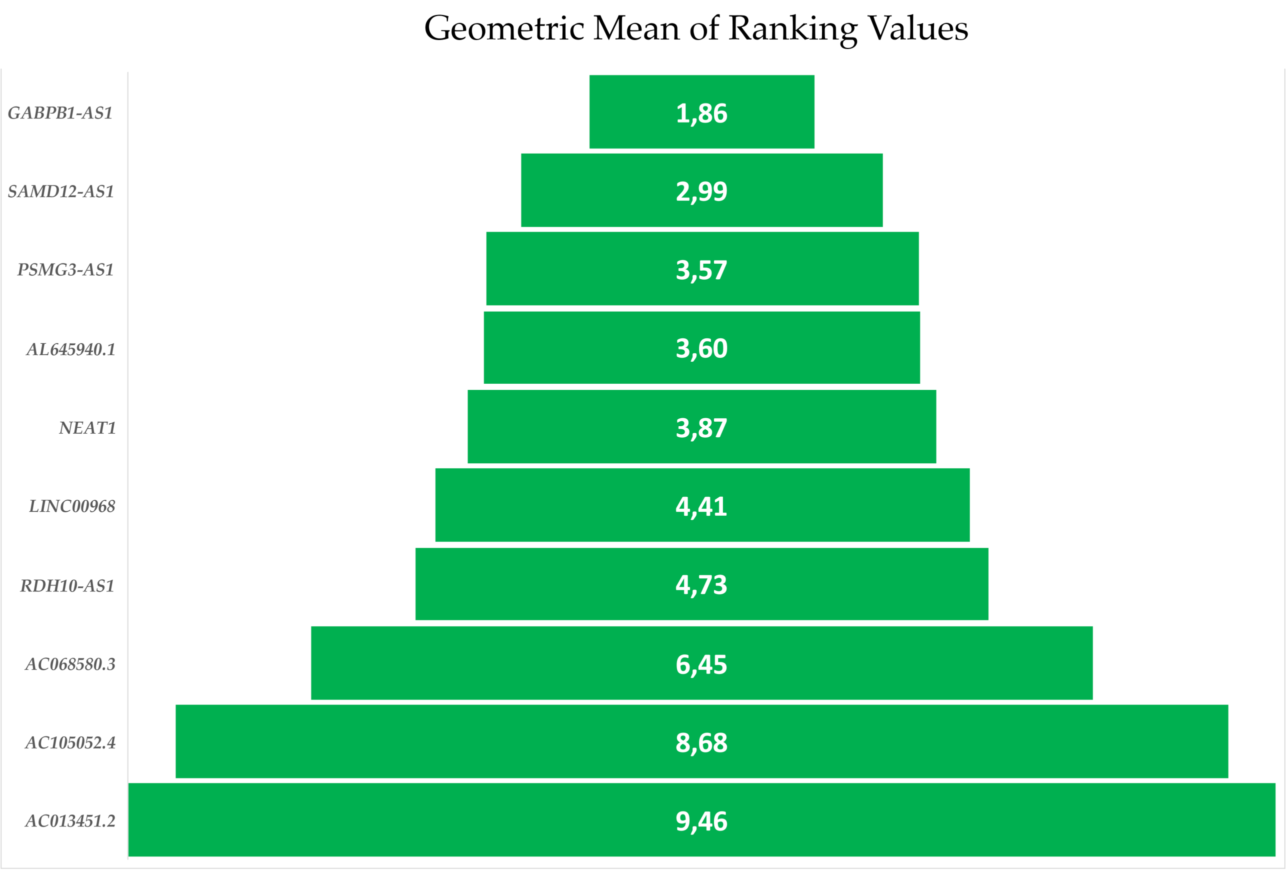

Figure S1. qRT-PCR selected lncRNAs stability results. The funnel chart shows the ranking of analyzed lncRNAs stability, obtained from the geometric mean of rankings values computed by Delta CT, GeNorm, NormFinder and BestKeeper algorithms. The lower stability value corresponds to more stably expressed gene.
